# Diabetes precision medicine: plenty of potential, pitfalls and perils but not yet ready for prime time

**DOI:** 10.1007/s00125-022-05782-7

**Published:** 2022-08-24

**Authors:** Simon Griffin

**Affiliations:** 1 MRC Epidemiology Unit, Institute of Metabolic Science, School of Clinical Medicine, University of Cambridge, Cambridge, UK; 2 Primary Care Unit, Department of Public Health and Primary Care, School of Clinical Medicine, University of Cambridge, Cambridge, UK

**Keywords:** Clinical epidemiology, Diabetes, Omics, Personalised medicine, Precision medicine, Public health, Review, Spectrum effect, Stratification, Type 2 diabetes

## Abstract

Rapid advances in technology and data science have the potential to improve the precision of preventive and therapeutic interventions, and enable the right treatment to be recommended, at the right time, to the right person. There are well-described examples of successful precision medicine approaches for monogenic conditions such as specific diets for phenylketonuria, and sulfonylurea treatments for certain types of MODY. However, the majority of chronic diseases are polygenic, and it is unlikely that the research strategies used for monogenic diseases will deliver similar changes to practice for polygenic traits. Type 2 diabetes, for example, is a multifactorial, heterogeneous, polygenic palette of metabolic disorders. In this non-systematic review I highlight limitations of the evidence, and the challenges that need to be overcome prior to implementation of precision medicine in the prevention and management of type 2 diabetes. Most precision medicine approaches are spuriously precise, overly complex and too narrowly focused on predicting blood glucose levels with a limited set of characteristics of individuals rather than the whole person and their context. Overall, the evidence to date is insufficient to justify widespread implementation of precision medicine approaches into routine clinical practice for type 2 diabetes. We need to retain a degree of humility and healthy scepticism when evaluating novel strategies, and to demand that existing evidence thresholds are exceeded prior to implementation.

**Graphical abstract:**

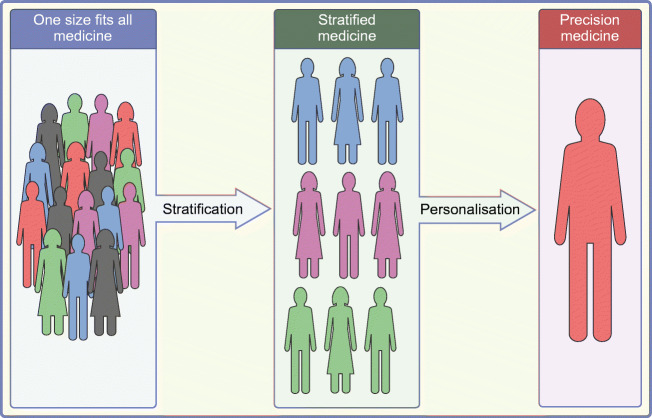

**Supplementary Information:**

The online version contains a slideset of the figures for download, which is available to authorised users at 10.1007/s00125-022-05782-7.

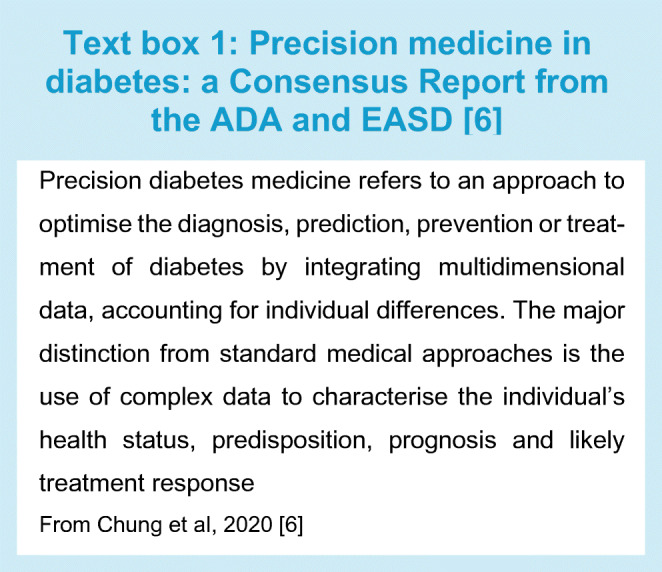

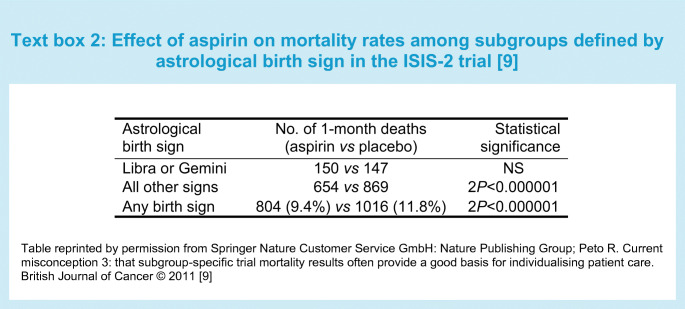

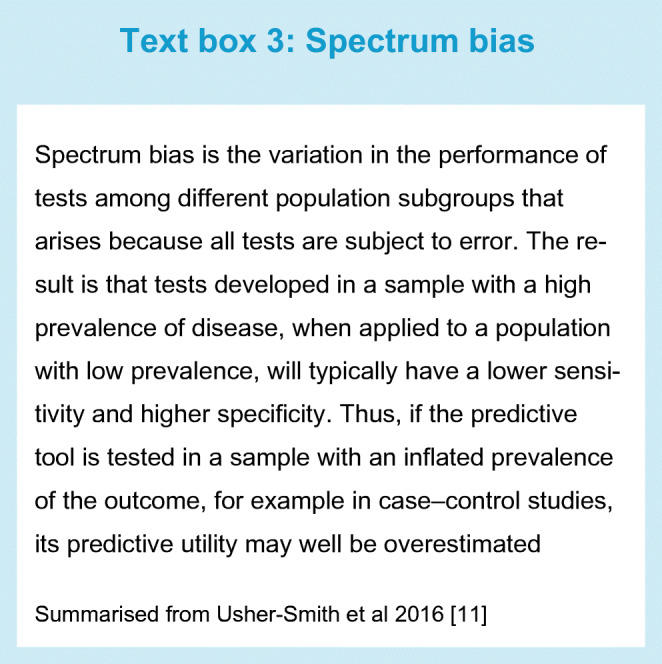







## Introduction

Rapid advances in technology and data science have the potential to improve the precision of preventive and therapeutic interventions, and enable the right treatment to be recommended, at the right time, to the right person. Type 2 diabetes is a multifactorial, heterogeneous, polygenic palette of metabolic disorders for which a ‘one size fits all’ approach is flawed [1]. However, we need to retain a degree of humility and healthy scepticism when evaluating novel strategies, and to demand that existing evidence thresholds are exceeded prior to implementation. In this non-systematic review I will highlight limitations of the evidence, and the challenges that need to be overcome prior to implementation of precision medicine in the prevention and management of type 2 diabetes. The polarised focus on limitations and challenges is deliberate, and serves as a counterpoint to the accompanying papers describing the rapid recent advances in the field, highlighting some opportunities for future research.

**Fig. 1:**
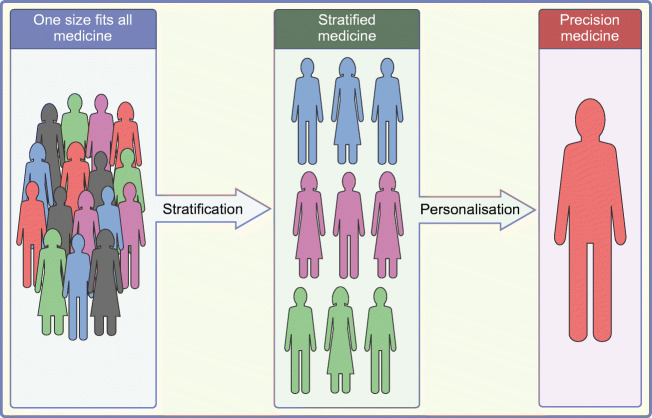
Stratified and precision medicine. This figure is available as part of a downloadable slideset

I have long advocated for stratification, having developed a diabetes risk score, derivatives of which are used in many diabetes prevention and early detection programmes [2]. More recently I have researched the potential for more nuanced cancer screening programmes based on simple phenotypic and/or genetic information, in order to minimise harms and maximise benefits by varying screening intervals and the age of onset of screening [3–5]. However, there is a material difference between a rough ranking of a population (stratification) and so-called precision medicine approaches to inform recommendations to individuals about disease prevention or treatment, for which evidence of effectiveness, let alone cost-effectiveness, is still lacking (Fig. 1).

The ADA–EASD Consensus Report definition of precision diabetes medicine is shown in Text box 1 [6]. The major distinction from standard medical approaches is the use of complex data to characterise the individual’s health status, predisposition to disease, prognosis and treatment response.

There are well-described examples of successful precision medicine approaches for monogenic conditions such as specific diets for phenylketonuria [7], and sulfonylurea treatments for certain types of MODY (see [8] as an example). However, the majority of chronic diseases are polygenic. It is unlikely that the research strategies used for monogenic diseases will deliver similar changes to practice for polygenic traits. Examples from cancers, where mutations in cancer tissue can inform treatment decisions, also do not translate well to diabetes because we do not have access to relevant patient tissue in the clinical setting.

I will discuss my concerns around precision medicine under four headings: (1) spurious precision; (2) unnecessary complexity; (3) personalised versus precision medicine; and (4) the individual.

## Spurious precision

Access to large datasets, omics technologies and artificial intelligence can lend a patina of precision to the science, for example by generating narrow confidence intervals, albeit around potentially biased and inaccurate estimates. The following examples serve to highlight why the precision is, thus far, frequently spurious.

Guidelines and treatment decisions are commonly based on average effects and overall evidence of cost-effectiveness of interventions among trial participants who, ideally, are similar to the patients that we care for. This evidence rarely extends to identifying individuals for whom the treatment will be particularly beneficial or harmful. Much of the science behind precision medicine is based on subgroup analyses. That is to say, we are led to believe that a subset of the sample, defined by a particular set of characteristics, experiences benefits or harms of a treatment much more or less than the rest of the population. Since this is predictable, the proponents of precision medicine believe that these characteristics should therefore influence which medication we prescribe to an individual.

Such subgroup analyses are, or should be, hypothesis-generating rather than forming the basis for changes to policy and practice. A classic example of the dangers of subgroup analysis comes from the ISIS-2 trial of aspirin after heart attack [9]. Overall, aspirin led to a highly significant 23% reduction in mortality at 1 month, but when stratified by astrological sign, because of the play of chance, the apparent effects differed from one subgroup to another. So while there was no apparent effect of aspirin among people with the star signs Libra or Gemini, aspirin appeared to reduce the mortality rate by 50% in people born under the star sign of Capricorn (see Text box 2) [9]. Trial subgroup claims are commonly unsupported by the trial data, sensitive to spurious or chance findings, and rarely corroborated [10].

There are limitations in the design and analysis of precision medicine studies. Much of the research is based on case–control studies, or features sampling from the extremes of the distribution of the exposure or the outcome. As measures of discrimination (sensitivity, specificity and area under the receiver operating characteristic [ROC] curve) are not independent of prevalence, the predictive utility of precision medicine characteristics is likely to be exaggerated due to spectrum bias (see Text box 3) [11]. Furthermore, even though precision medicine studies frequently concern prediction, for example of medication response or adverse effects, results are often expressed as odds ratios rather than measures of discrimination (such as sensitivity, specificity, predictive value, likelihood ratio or area under the ROC curve).

The contribution of precision medicine to behavioural approaches to the prevention and management of type 2 diabetes remains uncertain. Remission and prevention of type 2 diabetes are achievable via energy-restricted diets [12, 13]. In relation to remission, energy restriction appears to be more important than the specific content of the diet [14]. In relation to diabetes prevention, progression from impaired glucose tolerance to diabetes can be reduced by dietary modification targeting weight loss, regardless of genetic predisposition. Nevertheless, a lucrative, unregulated, precision nutrition industry has developed, supported by academic vested interest. The purported benefits of precision nutrition depend on a number of key assumptions: that different individuals have variable responses to the same nutrition intervention, that responses are reproducible, that responses can be understood and predicted by measurable characteristics such as omics, and that these characteristics (for example the microbiome) can be accurately specified with a single sample.

A series of detailed analyses of data from the PREDICT study has generated evidence of inter-individual variability in postprandial metabolic responses to standardised diets, that is associated with factors such as baseline sequencing of an individual’s gut microbiome rather than their human genotype [15, 16]. In addition, the PREDICT study has quantified the association between postprandial dips in glucose and hunger and subsequent energy intake [17]. However, Hall and colleagues have highlighted a number of issues that hamper interpretation of data from the continuous glucose monitoring (CGM) used in PREDICT. They have demonstrated intra-individual variation of postprandial glucose according to the CGM device used [18]. The inter-device variability could arise from the different anatomical locations used for each device, and hence the interstitial fluid sampled. However, it is unclear which interstitial fluid is the most relevant both physiologically, and in relation to risk of complications. Furthermore, identifying the signal from the noise is made more challenging by the variable responses within the same person, using the same device, to the same meal 1 week apart, even in a controlled metabolic laboratory (K.D. Hall, National Institute of Diabetes and Digestive and Kidney Diseases, Bethesda, USA, personal communication). Advocates of precision medicine highlight inter-individual variation to justify the need for bespoke diets, but pay less attention to this intra-individual variation, which adds complexity and increases uncertainty.

A personalised, rather than precision medicine, approach to supporting people with dietary change might consider an alternative set of correlates such as an individual’s psychological and environmental context [19, 20], which may be more influential determinants of habitual behaviours such as diet. Our understanding would be enhanced by more studies incorporating randomisation of individuals to the precision nutrition intervention or a standard care control group. Such studies are commonly required before regulatory approval, commissioning and implementation of health-related behavioural interventions. One such example reported significant, sustained (12 months) effects on CGM-determined glucose levels of a precision diet based on a machine learning algorithm that integrates clinical and microbiome features to predict personal postprandial glucose responses, compared with a Mediterranean diet, among people with ‘prediabetes’ [21]. In addition, effects were seen on HbA_1c_, for which the relationship with patient health outcomes has been well characterised. This single-blind explanatory trial was rigorously conducted, but inevitably generates additional questions. In particular, how much more effective in terms of diabetes prevention would such a precision diet intervention be compared with the existing ‘one size fits all’ diabetes prevention strategies focused on weight loss [13]? What were the key components of the complex dietary intervention? Adherence to the respective diets was assessed by self-report—is it possible that the persuasive, bespoke nature of the precision intervention led to better adherence to what appeared to be effectively a low carbohydrate diet? Greater progress has been made in other disease areas, for example psychiatry, in the trial evaluation of pharmacogenomic-guided treatment selection [22]. Precision medicine approaches involving pharmacogenomic testing for drug–gene interactions do indeed reduce prescription of medications with predicted drug–gene interactions. However, effects on patient outcomes such as symptom remission are small and short-lived [23].

Next there is the issue of intermediate endpoints. Precision medicine in diabetes has been narrowly focused on blood glucose levels, which have a relatively weak relationship with the main burden of type 2 diabetes, namely cardiovascular disease. Indeed, some medications that lower glucose, such as gliptins, have no effect on cardiovascular risk [24], whereas others, such as rosiglitazone, may increase risk [25]. We do not know how all of the drugs work, nor the mechanisms of all adverse effects. Furthermore, several drugs for type 2 diabetes reduce the risk of complications via mechanisms other than glucose lowering [26], so advising patients about which glucose-lowering drug to take, based on small genetic differences in glucose-lowering effects, is rather missing the point. Precision medicine advocates measure the easily measurable and base clinical recommendations on influencing the easily measurable. A personalised approach considers a wide range of potentially mutable determinants of disease and disease outcome, and incorporates a variety of biopsychosocial factors during shared decision-making concerning treatment options. There remains a lack of trial evidence that a precision medicine approach is more effective than existing strategies at improving outcomes deemed important by patients and clinicians.

Brilliant scientists, undertaking elegant studies, supported by rapidly advancing technology, have described the polygenic architecture of type 2 diabetes, common alleles with small, cumulative effects on disease risk. However, thus far these discoveries have had limited clinical utility in terms of disease prediction or prevention, and have made relatively small contributions to disease classification and precision medicine approaches to treatment. We need to be humble and acknowledge our ignorance when translating discoveries into recommendations for patients. Most single nucleotide polymorphisms (SNPs) are in non-coding regions of the genome. For the most part, we cannot link variants to biology and clinical endpoints. We do not know what tissues genetic variants influence, and at what stage in the disease trajectory the influence may be important. There are also few, if any, replicated examples of gene–environment interactions in diabetes aetiology that can be readily translated into specific advice to people, at sufficiently high absolute risks, to justify the effort [27–29]. Most of the omics precision medicine research has been based on data from populations of European ancestry, whereas the greatest burden of diabetes and its complications is in non-white populations, for which the evidence might not be relevant, even if the necessary technology were affordable and widely available [27].

A major, but rarely acknowledged, limitation of studies of effects and adverse effects of medication is that patients do not take their medicines as prescribed [30]. In genetic studies of treatment response or adverse effects, adherence is either not measured, or it is not measured with accuracy. In addition, genetic studies of treatment response often use trial datasets. Participants enrolled in trials have higher levels of adherence than patients in routine practice. Indeed, they often include a run-in phase prior to randomisation to ensure that this is the case. I will return to the theme of medication adherence in the subsequent sections. Precision medicine studies also ignore the placebo and nocebo effects, which have been well described for statins [31]. There is an order of magnitude difference in the reporting of adverse effects of statins among people who do and do not know that they are taking a statin. Medical record data include patients who are aware of the treatment that they have been prescribed (some of which they may actually ingest), in contrast with trial participants, who are usually unaware of the nature of their treatment. Medication adherence and placebo/nocebo effects will therefore affect the validity or the translation of findings from precision medicine studies to routine practice, or possibly both, and need to be considered when data are analysed and interpreted. Finally, while trials have limitations, in particular generalisability, they minimise the threat of confounding and selection bias when attempting to quantify treatment effects. In contrast, use of electronic health records will always be constrained by the risk of treatment indication bias and residual confounding, even after the use of techniques such as inverse probability weighting.

## Unnecessary complexity

Humans are beguiled by techno-optimism and would rather have a clever, new, bespoke solution than simply apply, more effectively, what we already know. The famous socialist GP, Julian Tudor Hart, described how managing conditions like diabetes involves doing simple things well, for large numbers of people, few of whom feel ill.

In all healthcare settings, many people are not receiving elements of care that are known to be both effective and cost-effective. In the UK this is clearly demonstrated in the UK NHS National Diabetes Audit [32] (https://www.diabetes.org.uk/professionals/resources/national-diabetes-audit). Small improvements in the delivery of care to the population will have far bigger impacts on health outcomes than precision medicine approaches for selected individuals. Further evidence is provided by the STENO-2 trial, in which relatively small changes in the intensity of treatment of multiple risk factors were associated with 50% reductions in the incidence of cardiovascular events and premature mortality [33, 34]. These effect sizes are an order of magnitude bigger than any seen in precision medicine studies of polygenic diseases. Clearly, there should be no false dichotomy. We must continue to develop and evaluate new strategies, but not at the expense of shifting the focus away from successful implementation of tried, trusted and effective approaches. Furthermore, prior to implementation, new strategies must be supported by similar rigorous standards of evidence that are equivalent to those demanded of current recommendations.

Tudor Hart was also the first to describe the inverse care law—the principle that the availability of good medical care tends to vary inversely with the needs of the population [35]. Precision medicine runs the risk of exacerbating inequalities given that, assuming it works, it will only be available to rich individuals, within high-income countries, for the foreseeable future.

The issue of unnecessary complexity also applies to a topic that I touched on in the previous section, medication adherence. For treatments with trial evidence of overall effectiveness, a key determinant of benefit for an individual is whether they actually take the medication or not. One factor known to reduce adherence is the presence of adverse effects. Precision medicine studies have sought to identify genetic markers of the risk of adverse effects in order to inform prescribing decisions. In one such case–control study, Dawed et al identified a SNP and concomitant medications associated with metformin side effects [36]. I did a back of the envelope calculation comparing those with and without adverse effects. The univariable association for sex was over 1.5 times greater than for the G allele for the intronic SNP *SLC29A4.* Thus, if you know the sex and the age of the patient, you get better prediction of the risk of adverse effects than you do from the SNP. The strength of the associations reported in the paper may be exaggerated due to the sampling strategy and study design. Nevertheless, the study highlights potential mechanisms underlying adverse effects and the fact that simple routinely available information may predict response to medication better than genetics. Furthermore, it is important to be aware of other well-known predictors of adherence, such as dosing regimen, patient beliefs, reminder systems, and communication with and trust in the prescribing physician [37].

## Personalised versus precision medicine

The finding that more complex omics information does not improve the predictive and clinical utility of simpler, routinely available information extends to the differential response to glucose-lowering treatment. Genetic predictors of drug efficacy with large effect sizes are likely to be rare [38]; compared with standard approaches genotype-guided treatment has not consistently increased effects and reduced adverse effects in other disease areas (for example treatment with tamoxifen and warfarin) [39], and genetic information may well increase costs, patient anxiety and inequalities [39]. Although, there are examples of genetic variants associated with glycaemic response [40], greater progress towards individualised treatment recommendations is being made using routinely available phenotypic data from electronic medical records and trial datasets [41]. Such an approach is perhaps closer to personalised than precision medicine, in that it integrates patient characteristics from multiple domains into the therapeutic decision-making process, albeit in a formal standardised statistical way. Personalised medicine has been practiced for centuries. As stated by Sir William Osler [42], ‘It is much more important to know what sort of a patient has a disease than what sort of disease a patient has’ and ‘The good physician treats the disease; the great physician treats the patient who has the disease.’

Most patients with diabetes have other comorbid conditions and hence take other treatments. The negotiation about diabetes treatment options needs to take this into consideration. I would argue that many important domains are not represented in electronic health records and trial datasets, for example those referred to in the section on adherence above. A personalised medicine approach involves education, negotiation and shared decision-making with patients, followed by monitoring of acceptability, side effects, adherence, quality of life and HbA_1c_ over the subsequent 10 weeks. New policies would need to demonstrate superiority over such a strategy prior to implementation.

## The individual

Among adults, when combined with routinely available information, polygenic risk scores do improve discrimination by a small amount, and hence identification of individuals at increased risk of diabetes. However, preventive interventions such as promotion of a healthy diet, physical activity and weight loss are nonspecific—they are beneficial for all high-risk individuals, and indeed, the majority of the population. Plus, they reduce the risk of a range of diseases other than diabetes, for example cardiovascular disease and cancers. Consequently, more precise targeting does not increase effectiveness but may improve efficiency, as the delivery of scarce resources more closely mirrors the population distribution of risk. However, the high absolute risk associated with obesity at any level of genetic risk underlines the importance of universal rather than targeted approaches to behavioural intervention [43].

Proponents of polygenic risk scores stress their value in identifying high-risk individuals from birth. The challenges of implementation are illustrated by considering the counterfactual: would you advise the parents of a baby with a low lifetime risk of diabetes that it is safe for their child to be sedentary, consume an unhealthy diet and become overweight?

The techno-optimists who promote precision medicine appear to believe that sharing the spuriously precise information from omics technologies with patients will have a far more powerful impact on their behaviour than existing risk information. However, we have shown in trials [44, 45], and a systematic review and meta-analysis [46], that this is not the case. The key health behaviours related to diabetes and its complications, such as diet and physical activity, are not affected by the provision of genetic or phenotypic risk information [44]. Instead, as highlighted by William James (the father of American behavioural science), these behaviours are largely habitual, automatic, unaccompanied by conscious reflection and cued by environmental stimuli [47].

The main burden of diabetes is cardiovascular, and in common with blood pressure and cholesterol, glycated haemoglobin exhibits an approximately normal distribution and a linear association with the risk of cardiovascular disease [48]. The prevention paradox described by Geoffrey Rose demonstrates that, in this situation, more cardiovascular events would be prevented by shifting the population distribution of glucose to the left, than by targeting the minority of individuals close to or above the diagnostic threshold for diabetes [49]. There is a greater need for scalable interventions targeting the individual and collective determinants of hyperglycaemia, than for precision medicine initiatives for rich individuals in high-income countries. Not only is the former likely to be more effective, but as it makes fewer demands on the agency of individuals, is less likely to increase inequalities [50].

**Fig. 2:**
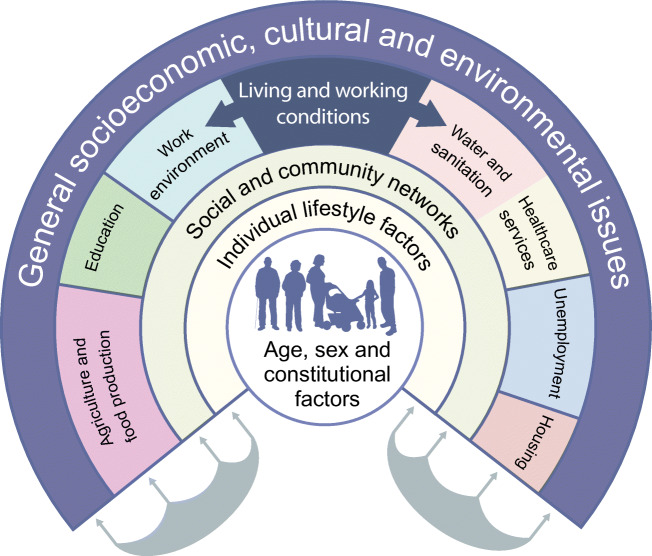
The main determinants of health. Adapted from Dahlgren G, Whitehead M, 2007 [54, 55]. This figure is available as part of a downloadable slideset

Healthcare has a marginal impact on population health. Type 2 diabetes is as much a societal problem as a medical one. As Rose also pointed out, the primary determinants of disease are mainly economic and social, and therefore the remedies must also be economic and social [51]. Instead of focusing down on the molecules and cells within individuals, we should be lifting our gaze upwards to the more important individual and collective determinants of the diabetes pandemic (Fig. 2). These determinants range from individual knowledge, attitudes and beliefs, portion sizes, internal and external built environments, accessibility of healthy and ‘takeaway’ food, through to government food, transport and trade policies, and the activities of the multinational producers of processed foods, which have a profound influence on global health.

Governments and research funders appear to find the allure of bespoke, technical, science-based solutions that target individuals, such as precision medicine, beguiling. In contrast, most administrations are more comfortable on the lower rungs of the Nuffield ladder of state interventions (leaving decisions up to individuals, informing the public and engaging with industry), and resist demands to adopt policies on the higher rungs (regulation, taxation, restricting choice and banning) [52], irrespective of the underlying evidence. In his 2015 State of the Union Address, Barack Obama unveiled details of the ‘Precision Medicine Initiative’ and set aside $215 million to fund research [53]. I am not aware of an equivalent amount being made available to support public health research.

## Conclusion

Precision medicine represents an exciting field of scientific enquiry with the potential to revolutionise clinical practice. Unfortunately, successful implementation in the field of type 2 diabetes is limited by the heterogeneous, polygenic nature of the condition. Many precision medicine approaches are spuriously precise, overly complex, and too narrowly focused on blood glucose levels and on the individual and not their context. The evidence to date is insufficient to justify widespread implementation of precision medicine approaches into routine clinical practice. Strategies to inform selection of glucose-lowering medication that are based on routinely available phenotypic data show promise, but need more trial evaluation and should consider medication adherence. Our focus should continue to be on improving the application of personalised medicine, treatments and policies that are known to be helpful and cost-effective, on average, for a wide range of patients and populations. In developing and evaluating precision medicine it is important to see the new approach as an addition to the more traditional personalised medicine. More trials are needed to increase understanding, such trials should extend assessment to include non-glycaemic outcomes such as other cardiovascular risk factors and quality of life. Disease prediction models and new medications are subject to regulation, and precision medicine approaches should follow suit.

## Supplementary information

ESM 1 (PPTX 392 kb)

## Abbreviations

CGM: Continuous glucose monitoring
ROC: Receiver operating characteristic
SNP: Single nucleotide polymorphism
