# Supplementary material for: Diabetes precision medicine: plenty of potential, pitfalls and perils but not yet ready for prime time
Source: Diabetologia. 2022 Aug 24;65(11):1913–21. doi: 10.1007/s00125-022-05782-7 (PMC9522689; doi:10.1007/s00125-022-05782-7)
Supplement: Supplementary file 1 — (PPTX 392 kb) [file 125_2022_5782_MOESM1_ESM.pptx]

## Slide 1
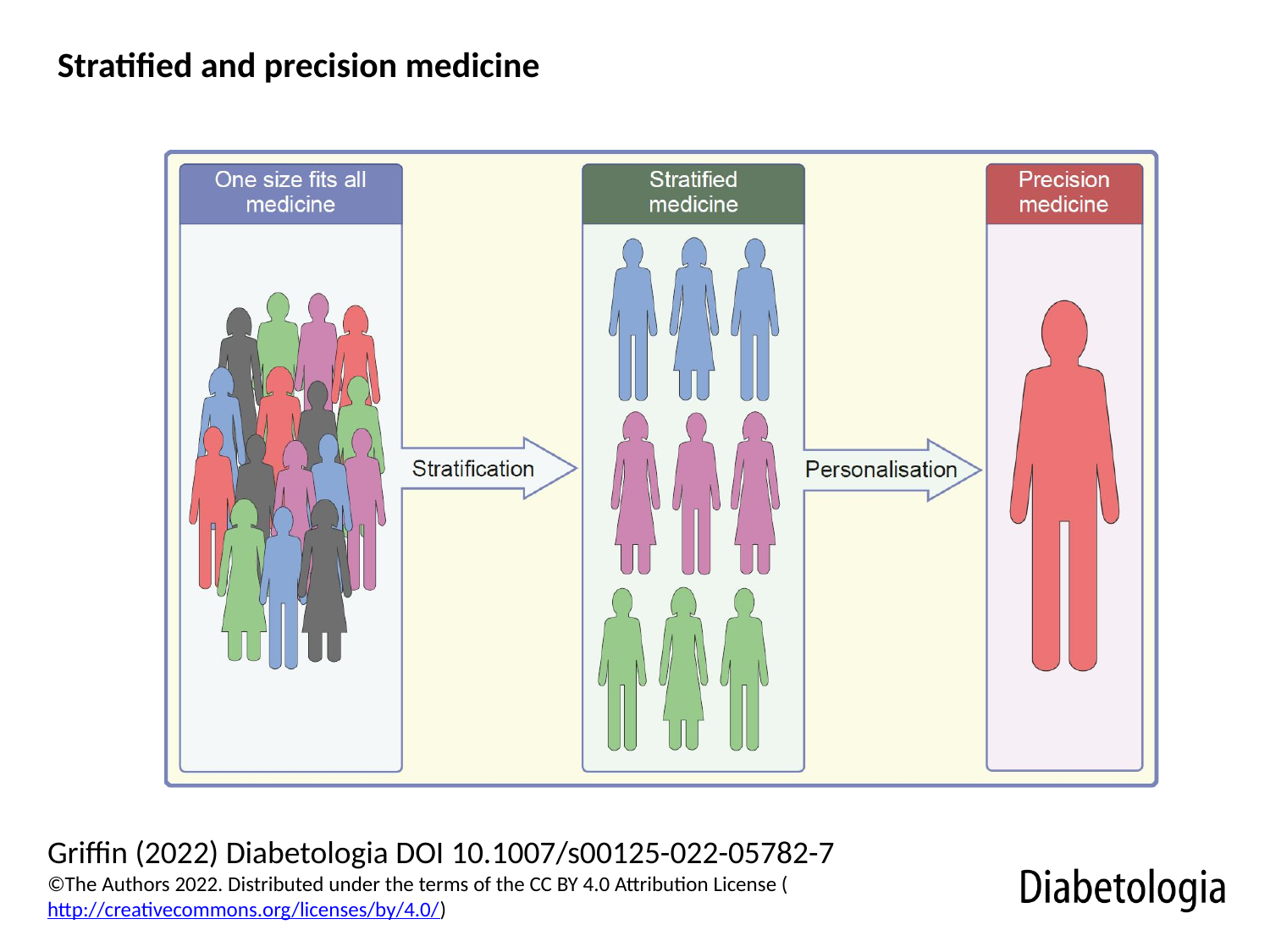

Stratified and precision medicine
Griffin (2022) Diabetologia DOI 10.1007/s00125-022-05782-7
©The Authors 2022. Distributed under the terms of the CC BY 4.0 Attribution License (http://creativecommons.org/licenses/by/4.0/)

## Slide 2
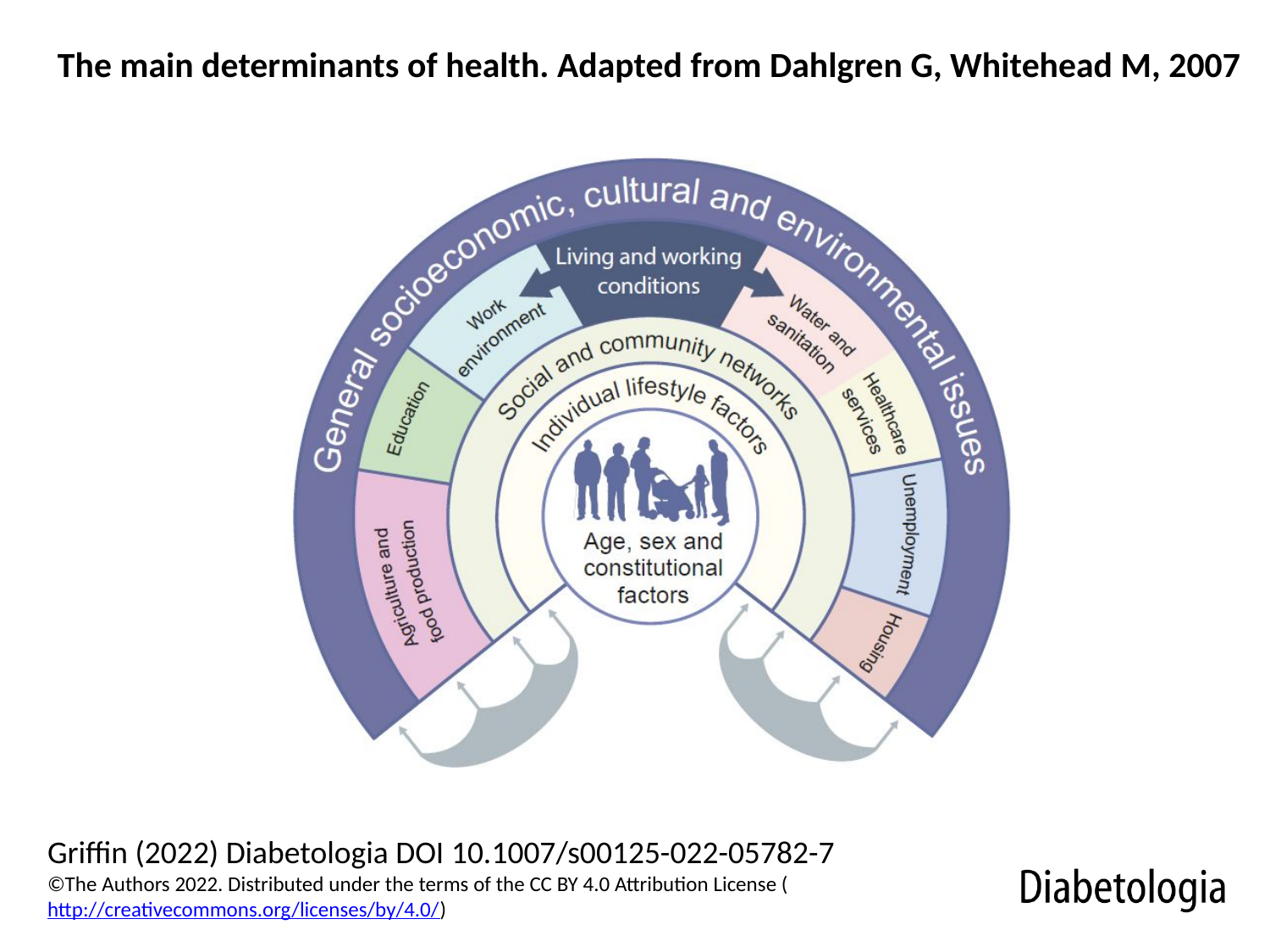

The main determinants of health. Adapted from Dahlgren G, Whitehead M, 2007
Griffin (2022) Diabetologia DOI 10.1007/s00125-022-05782-7
©The Authors 2022. Distributed under the terms of the CC BY 4.0 Attribution License (http://creativecommons.org/licenses/by/4.0/)
